# Computational method for multiphase flow characterization in the gas refinery

**DOI:** 10.1016/j.heliyon.2020.e03193

**Published:** 2020-01-18

**Authors:** Abolfazl Varvani Farahani, Mohsen Montazeri

**Affiliations:** Department of Electrical Engineering, Faculty of Electrical Engineering, Shahid Beheshti University, Tehran, Iran

**Keywords:** Petroleum engineering, Soft computing, Control systems, Nonlinear control system, Computational fluid dynamics, Petroleum industry, Decentralized measurement system, Multi-phase flow, Condensate and gas, Soft sensor, Nonlinear observer, Mean value theorem, Unscented Kalman filter

## Abstract

This paper presents a new computational method for the decentralized multiphase flow measurement based on the interconnections between the two subsystems to precisely estimate the states of the multiphase flow at the gas refinery. The states of the condensate and gas sub-systems were separately estimated using the Differential Mean Value Theorem by considering the relationship between two subsystems, designing an observer and converting the conditions to linear matrix inequality. To check the stability and performance of the system against the changes, the Lyapunov theory has been used. The states behavior investigated with and without disturbance in the system output and dynamics. Additionally, the Unscented Kalman Filter based on the simplified drift flux model was used to estimate the states. It is found that both observers are capable to identify the states with some differences in performance and drift flux model is sufficient for estimation of parameters and states.

## 1 Introduction

**Figure 1:**
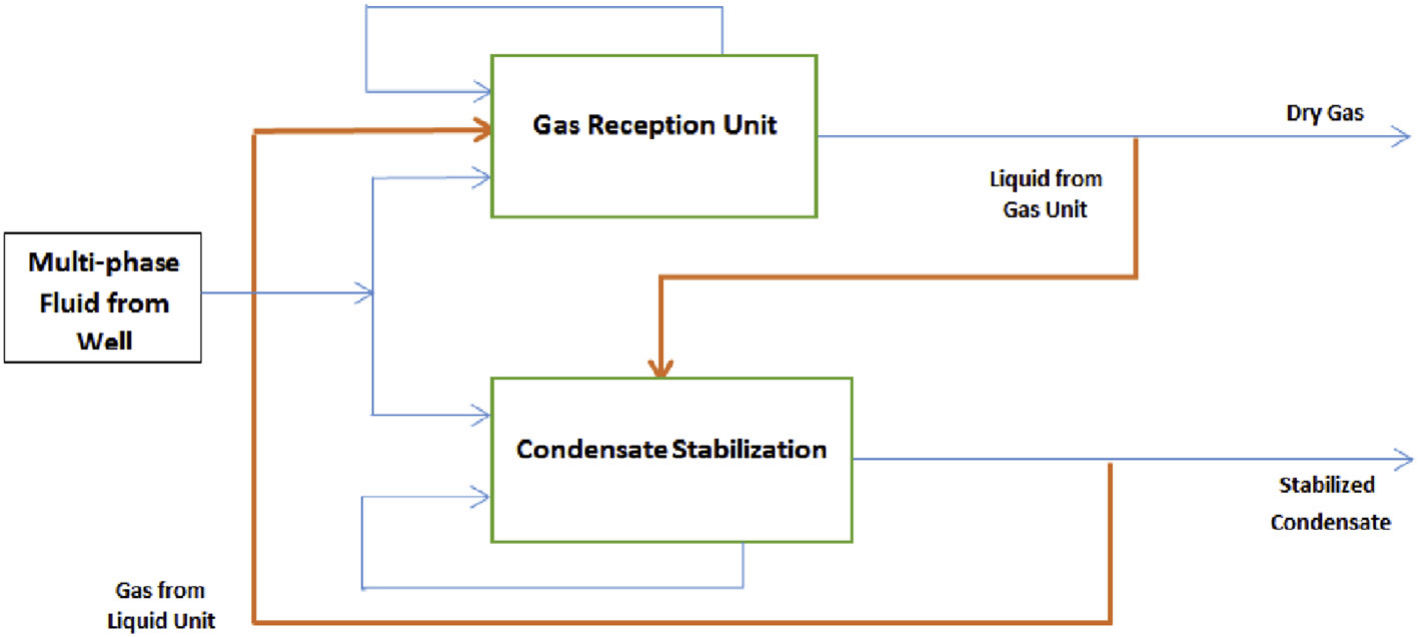
Existing subsystems, the system boundaries, and the inlet and outlet of the subsystems.

**Figure 2:**
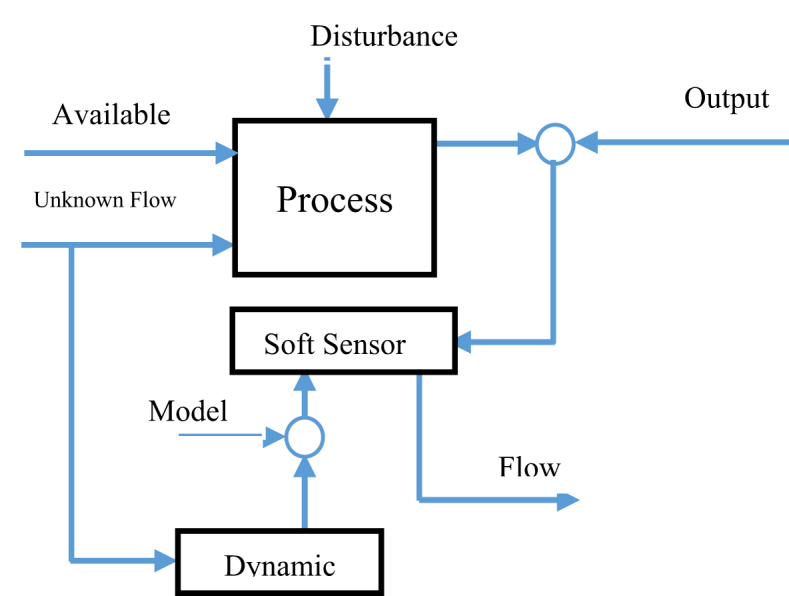
Schematic of Multi phase flow soft sensor.

The growth of population and limitation of hydrocarbon sources in the world have resulted in higher demand which, in turn, continuously affect the price of these products. High price of energy resources, including oil and gas extracted from the well may be processed at the platform, or may be directly oil and gas, depicts the need for increased measurement precision. Although technological progresses in the field of single-phase fluid measurement have been acceptable and the existing systems are adequately accurate, they are not precise enough when are used for multiphase fluids (gas, liquid, solid). Due to the nature of the fluid extracted from the well and high fluid temperature and pressure variations over the long transportation pipeline, it may behave like a multiphase fluid. Therefore, it should be measured as a multiphase fluid either at the platform or the entrance of the refinery. Figure 1 presents a schematic of the input multiphase fluid (gas-liquids), distribution among different refinery units, and existing measurements (see Figure 2).

In the recent years, the multiphase fluid measurement has attracted many researchers, and many studies have been conducted on different aspects and applications of it. Some researchers have used single phase conventional technologies to measure multiphase flow rate [1, 2, 3, 4], but due to the strong dependence of flow measurement on the composition and flow characteristics in two-phase fluids, these methods have a high level of measurement uncertainty. Some papers review different instruments, measurement strategies and principle technologies introduced until now and describes some methods such as inferential approach, phase separation, Microwave attenuation, Impedance method, gamma attenuation, cross-correlation method for measuring the velocity and phase fraction in multiphase flow in pipeline [5, 6, 7]. In [8] the mass flow rate is estimated in a gas–liquid two-phase flow through a measurement system consisting of a Venturi or orifice plate flow meter coupled to an impedance void meter of the non-intrusive resistance type. In [6, 7, 9] pulsed-array sonar clamp-on flow meters as the primary flow measurement devices have been coupled to an Equation of State based compositional model to facilitate the use of composition data. Some studies focus on using the non-intrusive instrumentations for estimation of multiphase flow specifications, for example, a study reviews the using, limitations and future developments of non-intrusive optical infrared sensing technique for gas–liquid flow characterization in pipes [10], and authors of [11] studied the effects of high viscosity on heavy oil two-phase flow characteristics such as pressure gradient, liquid holdup, slug frequency and slug liquid holdup using a non-intrusive advance instrumentation such as Electrical Capacitance Tomography. This technique is widely used for gas-solid two phase flow measurement but suffers from the nonlinear response of the measurement to the particle distribution [12, 13].

Due to the technological challenges, the conventional methods either are costly and need much equipment, or are not capable of making highly-accurate direct measurements. As a result, using soft sensors is a solution to overcome the existing problems and increase the accuracy of multiphase fluids measurement [14]. In this regard, the Different types of Kalman filter (KF) such as extended (EKF) or unscented (UKF) Kalman Filter area well-established technique for state estimation [15, 16].

As a result, a broad range of studies have concentrated on the application of inferential methods with a focus on wells subjected to gas lifting operation [17, 18, 19]. In [20], assuming that the lateral flows of the porous tubes are known, the volume fraction and different phase velocities were investigated. Despite numerous researches on the multiphase flow and soft sensors, the most attention has been concentrated on the multiphase fluids for drilling, gas lifting, and down-hole systems rather than multiphase flows in production units, refineries, and petrochemical plants [21, 22]. In most oil and gas platforms and production units like gas refineries, due to the fluid nature, lack of appropriate technology, and the high cost of the peripheral equipment, the direct measurement of the outlet fluid from the well is neither accurate nor even feasible.

The simplified drift flux model (DFM) is based on one momentum conservation equation, allows for transition between single and two phase flows, without predictions of flow-regime. In this model, multiphase fluid treats as a mixture but at any moment accounts an empirical slip law for the velocity difference between gas and liquid. The drift flux model (DFM) has been shown to typically have a better performance than other models for multiphase fluids [23, 24, 25]. In this paper, a simplified DFM is used.

Despite many studies on the multiphase flow measurement and soft sensors, there are scant studies on the flow measurement inside the refineries and petrochemical plants; therefore, this study intends to investigate this subject. Some studies have used DMVT capability in conversion of estimation error dynamics into linear parameter-varying (LPV) systems to ensure the validity of $\frac{\partial V}{\partial t}<0$ for the ordinary Lyapunov function, $V\left(x\right)=x^{T}Px$, which uses LMI to prove the convergence of the observer [26]. The existing flow measurement systems in a gas refinery's entrance have a high level of error and this low accuracy causes a large difference between the downstream and upstream flow measurement figures. Oil and gas production units are usually heavily instrumented with a large number of single/multiphase flow meters which have high establishment, maintenance and repair costs. For these reasons, soft sensors have been established as a serious powerful alternative to the traditional means in the process control of oil and gas fields [27]. Accordingly, this study intends to find a soft sensor to estimate multiphase fluids for two purposes: as a hot redundant system for the existing system and as a substitute for low-accurate available systems.

Some models have focused on monitoring of wells and parameter estimation for drilling, gas injection and gas lifting operations [17, 20] or they have used for parameter estimation and prediction of the primary variables in a single independent process like distillation towers, ammonia synthesis or reactors [28, 29]. But none of them are based on decentralized measurement and multiphase flow at the gas refinery's entrance considering the interconnections between plants.

The main contribution of this paper is, **presenting a new form of the soft sensor using the new decentralized Lyapunov-based observer to measure the multiphase flow at the entrance of a gas refinery, which leads to better performance, higher accuracy, and repeatability.**

In this paper, to show the performance of the proposed adaptive observer, it is evaluated against and compared with the Unscented Kalman Filter and HYSYS simulator using information collected from the actual process in a real gas refinery. Furthermore, DFM for flow characterization in the decentralized multiphase flow measurement system is evaluated.

The outstanding achievements accomplished with this soft sensor are as follows:

- This soft sensor increases accuracy of multiphase flow measurement by using a relatively simple model, without the need to use the costly and high-maintenance multiphase measurement systems.
- According to the API and AGA standards, acceptable accuracy of single phase fiscal flow measurement for gaseous is 1% and for liquids is 0.15%, using this soft sensor, RMSE metric for the liquid flow measurement is better than 0.12% and for the gas flow measurement is better than 0.34%. Thus, this technique not only can be used as an alternative to the costly multiphase measurement systems or low-accuracy conventional systems but also serves as a fault-detection system or as a backup when the existing measurement system is in fault or removed due to maintenance or replacement.
- Evaluating the DFM for flow characterization in the decentralized multiphase flow measurement system consisting of two interconnected subsystems in the gas refinery inlet employing a decentralized Lyapunov-based observer and Unscented Kalman Filter.
- Consistency of both estimators with the real process in a gas refinery in the South Pars Gas Complex (SPGC) in Iran is shown.

## 2 Gas refinery system

Most inlet flows to the gas refineries, consist of multiphase hydrocarbon fluids. The overall system comprises two large interconnected sub-systems. The condensate stabilization unit and gas reception unit are two main sub-systems of the gas refinery's processing plant. The existing subsystems, the system boundaries, and the inlet and outlet of the sub-system are as shown in Figure 1.

In this system, the multiphase fluid passes through a slug catcher immediately after entering the gas refinery. Given the fact that condensate is not extracted completely from gas, so outlet condensate from the slug catcher contains some gas and outlet gas from the slug catcher contains some condensate. For this reason slug catcher output is divided into two two-phase lines (a gas line containing some condensates and a condensate line containing some gas). It is evident that the main system was assumed as a decentralized system composed of two distinct interconnected sub-systems. The simplified drift flux model (DFM), as a representation of the law of conservation of mass and momentum, are used to handle multiphase fluid in a tube. The sub-systems are described separately using three equations (conservation of mass for the liquid, conservation of mass for the gas, and conservation of momentum for the mixture). Consequently, two sets of three equations are obtained.

In practice, input flow to the refinery is obtained from the measurement of the refinery's output gas. In that, the dry gas delivered to the export pipeline from the refinery output is measured with a single-phase flow meters, then, the amount received at upstream is calculated by the application of shrinkage factor. For example, the measured amount of dry gas was 46.8 million-square-feet per day (MMSCFD), and by applying the shrinkage factor (in this case is equal to 1.13), received gas from the upstream is reported as 52.88 MMSCFD. In contrast, the well production at upstream was reported as the amount of gas delivered to the plant based on the presence of a high-pressure Venturi meter and/or based on the opening of the wellhead Choke valve. According to above table, this production was reported 57.9 MMSCFD. This method produced an error rate of 8% in the upstream-downstream exchanges, indicating a high level of error and low level of accuracy. The main causes of the errors were:

• Varying amounts of water, flare, fuel, etc. were assumed to be fix and certain.
• Reported shrinkage factor dated back to the refinery's commissioning time; whereas, all of the process conditions have been changed, equipment's efficiency reduced, therefore shrinkage factor could not be assumed fix
• The lack of accuracy of upstream meter and choke valve opening figures.
• Multiphase flow at the upstream and refinery's entrance.

The mentioned items are the clear reasons for using soft sensors to reduce energy dissipation and increase condensate and gas measurement accuracy (see Table 1).

**Table 1:** Input and output balance in real.

(MMSCFD)Values	Title
57.9	sent reach gas (upstream)
52.88	Received reach gas (refinery)
46.8	Export gas to IGAT
2.11	Condensate
1.97	Fuel
0.62	Acid gas to sulfur production
0.13	Flare
0.2	Water content

## 3 Dynamic model

The simplified drift flux model (DFM) are the main governing equations for flow in the pipe and law of conservation of mass and momentum have been used in dynamic models extraction in this study. This equations have been written for each subsystem separately in a way that for each subsystem, 3 equations are generated which are conservation of mass for liquid and gas and conservation of momentum for mixture and consequently, we will have 2 sets of a triple equation [20]. *One-dimensional equations* have been investigated by several researchers [30, 31, 32]*,* due to the high ratio of length/diameter, negligible motion in the directions perpendicular to the flow axis, the flow direction is dominating and because the flow velocity profile is fully developed, so by carrying out averaging of Navier Stokes equations at each cross section, one-dimensional equations are obtained.

Some assumptions must be considered before writing equations:

1. Condensate and water will be considered one phase liquid.
2. The flow velocity will be considered only in the flow direction and in any other direction it will be ignored.
3. Phase transfer is assumed ignorable.
4. Subsystems interconnections assumed to be single phase never include any second-phase.

The system dynamic model is extracted from the simplified drift flux model (DFM) and finally by generalizing it into two subsystems, a solvable general format will be achieved. The conservation of mass and momentum in a control volume will be as follows [33]:

(1) $$\frac{\partial \left(\rho \text{α }\right)}{\partial t}+\frac{\partial \left(\rho \text{αu}\right)}{\partial s}=\text{Φ}+m$$

(2) $$\frac{\partial \left(\rho \text{αu}\right)}{\partial t}+\frac{\partial \left(\rho \text{α}u^{2}\right)}{\partial s}=-\alpha \frac{\partial P}{\partial s}+\tau_{w}\frac{S}{A}+\phi +\rho g\alpha \sin\theta$$

In these formula, p is the line pressure, ρ is the density, α is the volume fraction of each phase, u is the velocity and t shows the time and s is the length of pipe, τ_w_ is the shear stress between surfaces, φ is the interfacial interaction, $S_{fr}$ is the friction losses and θ is the pipe inclination angle and m is the mass transformation degree and Φ is the mass flow from external source. The phase transfer in the interconnected lines is considered zero and the mass flow from external source is equal to the flow in the interconnections between subsystems.

(3) $$\sum \left(\frac{\partial \left(\rho_{i}\alpha_{i}\right)}{\partial t}+\frac{\partial \left(\rho_{i}\alpha_{i}u_{i}\right)}{\partial s}\right)=h_{lG}\;i=1,2$$

(4) $$\sum \left(\frac{\partial \left(\rho_{j}\alpha_{j}\right)}{\partial t}+\frac{\partial \left(\rho_{j}\alpha_{j}u_{j}\right)}{\partial s}\right)=h_{gL}\;j=1,2$$

where *L, G* indexes are used for condensate and gas subsystems respectively, $h_{lG}$ and $h_{gL}$ are the relation between liquid and gas subsystems in a way that $h_{lG}$ is the volume of extracted liquid from the gas subsystem to liquid process and $h_{gL}$ is the volume of extracted gas from the liquid subsystem to gas process. $h_{i}$ is directly measurable by primary measuring elements such as orifice and it includes the following specifications:

1. h_i_ can be measured directly and has limited norms and it is Lipschitz, in other words:

(5) $$a\leq \frac{\partial h_{i}(v\left(t\right))}{\partial v_{i}}\leq \text{b}$$

$$\text{a}=min_{z\in R^{n}}(\frac{\partial h_{i}\left(v\left(t\right)\right)}{\partial v_{i}}\left(z\right)),\;b=max_{z\in R^{n}}(\frac{\partial h_{i}\left(v\left(t\right)\right)}{\partial v_{i}}\left(z\right))$$

2. Interconnections between subsystems are single-phase and do not have second phase.
3. There is no return from the counter process of h_i_ and it is one way flow.
4. Considered disturbances are bounded and $\omega \left(t\right)\in L_{2}^{s}$.

Considering the limited states of the system, the nonlinear behavior of equations are Lipschitz with definite and limited range.

The law of conservation of momentum will be written for the mixture of each pipe. L, G indexes are used for different subsystems. L stands for condensate and gas mixture in liquid line of unit 103 and G stands for the mixture of condensate and gas in gas line of unit 100.

(6) $$\frac{\partial \left(\alpha_{L}\rho_{L}u_{L}\right)}{\partial t}+\frac{\partial \left(\rho_{L}\alpha_{L}u_{L}^{2}\right)}{\partial s}+\frac{\partial \left(\alpha_{\mathrm{lg}}\rho_{\mathrm{lg}}u_{\mathrm{lg}}\right)}{\partial t}+\frac{\partial \left(\rho_{\mathrm{lg}}\alpha_{\mathrm{lg}}u_{\mathrm{lg}}^{2}\right)}{\partial s}=-\frac{\partial p_{L}}{\partial s}-S_{\mathrm{frL}}$$

(7) $$\frac{\partial \left(\alpha_{G}\rho_{G}u_{G}\right)}{\partial t}+\frac{\partial \left(\rho_{G}\alpha_{G}u_{G}^{2}\right)}{\partial s}+\frac{\partial \left(\alpha_{\mathrm{gl}}\rho_{\mathrm{gl}}u_{\mathrm{gl}}\right)}{\partial t}+\frac{\partial \left(\rho_{\mathrm{gl}}\alpha_{\mathrm{gl}}u_{\mathrm{gl}}^{2}\right)}{\partial s}=-\frac{\partial p_{G}}{\partial s}-S_{\mathrm{frG}}$$

In the aforementioned conservation of momentum equations, the first equation is for the existing liquid and gas in the condensate line plus the inlet liquid of the interconnection of subsystems from gas process and the second equation is for the existing gas and condensate in gas line plus the received gas from the condensate subsystem.

As is seen in the left side of the equation, $S_{fr}$ exists instead of pipe shear stress. As regards to the fact that most of the pipe friction relations are based on empirical curves to solve this equation, an accurate model of friction loss must be presented [20]:

(8) $$S_{\mathrm{fr}}=\frac{f}{2d}\rho_{m}u_{m}^{2}$$

where d is the pipe diameter and f is the friction factor which depends on pipe roughness and Reynolds number which Blasius correlation has been used for that [34]:

(9) f=0.3164Re0.25

(10) Re=d.V.ρμ

where d is the pipe diameter, V stands for velocity, ρ represent density and μ is viscosity. The mixture velocity and density are as follows:

(11) $$\rho_{m}=\rho_{g}\left(1-\alpha_{l}\right)+\rho_{l}\alpha_{l}$$

(12) $$u_{m}=\alpha_{l}u_{l}+\alpha_{g}u_{g}$$

Flow model is complemented by algebraic slip law so slip relation is taken in to account for difference between gas and liquid velocities and drift relation is taken into account for difference between gas and mixture velocities. Algebraic slip and drift is as follows [35]:

(13) $$u_{g}=C_{0}u_{m}+u_{drift}$$

(14) $$u_{drift}=0.35\sqrt{\frac{gd\left(\rho_{l}-\rho_{g}\right)}{\rho_{l}}}$$

(15) $${C_{0}=1.2-0.2\left(\rho g/\rho l\right)}^{0.5}$$

For viscosity, there are several models and the most famous one is Einstein [7]:

(16) $$\mu_{m}=\mu_{l}\alpha_{l}+\mu_{g}\alpha_{g}$$

The gas pressure will be obtained as follows:

(17) $$P=\rho_{g}R_{g}T_{0}$$

Gas is a compressible fluid and its density depends on the pressure and temperature. In this study, since temperature is assumed to be constant, gas density will depend on pressure and relation (17) will be used.

The important point to note is that the pressure gradient in the right side of the momentum equation will be considered for the total pressure of mixture which is possible for homogenous distribution, but for multi-fluid model using one pressure for all the mixture is not true and may cause inconsistency. Considered model for this study is based on the mentioned model in [7] and will be as follows:

(18) $$U_{1}={\left(\begin{array}{c} \rho_{l}\alpha_{l}\\ \begin{array}{c} \rho_{g}\alpha_{g}\\ \left(\rho_{l}\alpha_{l}u_{l}+\rho_{g}\alpha_{g}u_{g}\right) \end{array} \end{array}\right)}_{Liquid}$$

(19) $$U_{2}={\left(\begin{array}{c} \rho_{l}\alpha_{l}\\ \begin{array}{c} \rho_{g}\alpha_{g}\\ \left(\rho_{l}\alpha_{l}u_{l}+\rho_{g}\alpha_{g}u_{g}\right) \end{array} \end{array}\right)}_{Gas}$$

(20) $$F_{1}={\left(\begin{array}{c} \rho_{l}\alpha_{l}u_{l}\\ \begin{array}{c} \rho_{g}\alpha_{g}u_{g}\\ \left(p_{L}+\rho_{l}\alpha_{l}u_{l}^{2}+\rho_{g}\alpha_{g}u_{g}^{2}\right) \end{array} \end{array}\right)}_{Liquid}$$

(21) $$F_{2}={\left(\begin{array}{c} \rho_{l}\alpha_{l}u_{l}\\ \begin{array}{c} \rho_{g}\alpha_{g}u_{g}\\ \left(p_{G}+\rho_{l}\alpha_{l}u_{l}^{2}+\rho_{g}\alpha_{g}u_{g}^{2}\right) \end{array} \end{array}\right)}_{Gas}$$

(22) $$Q_{1}={\left(\begin{array}{c} 0\\ 0\\ -S_{frL} \end{array}\right)}_{Liquid},\;Q_{2}={\left(\begin{array}{c} 0\\ 0\\ -S_{frG} \end{array}\right)}_{Gas}$$

(23) $$h_{1}={\left(\begin{array}{c} {\text{h}}_{lG}\\ 0\\ 0 \end{array}\right)}_{Liquid},\;h_{2}={\left(\begin{array}{c} 0\\ {\text{h}}_{gL}\\ 0 \end{array}\right)}_{Gas}$$

(24) $$\frac{\partial U_{i}}{\partial t}+\frac{\partial F_{i}}{\partial s}=Q_{i}+h_{i}\;\text{i}=\;1.2$$

where *U*_*i*_ is the state variable vector, *F*_*i*_ is the flux, *h*_*i*_ is the interconnections of subsystems and *Q*_*i*_ is the source term, *i* stands for various phases of flow and n is the number of existing phases, which is n = 2 in the present study and for this reason instead of using number, g or G is used for the gas phase and l or L is used for liquid phase.

As shown, pressure, velocity and phases volume fraction are state variables:

(25) $$\begin{array}{l} X_{g}={\left[\;P_{\mathrm{iG}}\;u_{G}\;\alpha_{\mathrm{lG}}\right]}^{T}\;\\ X_{L}={\left[\;P_{\mathrm{iL}}\;u_{L}\;\alpha_{\mathrm{lL}}\right]}^{T} \end{array}$$

Available measurements are pressure sensors, refinery single-phase meters (that measure gas and liquids as a single-phase with high accuracy), existing analyzers and also inlet meters of refinery that have high uncertainty. The accuracy of existing systems for measuring single-phase gas and liquids located in the refinery outlet is based on MPMS section of API standard and is better than 0.15%, but the existing measuring system in the refinery inlet has more than 5% error which can just be applied for operational purpose, because of using orifice systems for multiphase liquid.

(26) $$\begin{array}{l} y_{g}={\left[P_{\mathrm{iG}}\;u_{\mathrm{Gout}}\;G_{\mathrm{out}}\;\right]}^{T}\\ y_{L}={\left[\;P_{\mathrm{iL}}\;u_{\mathrm{Lout}}\;L_{\mathrm{out}}\right]}^{T} \end{array}$$

The model inputs are the subsystems interconnections which in this case are the amount of gas from the liquid subsystem to the gas production unit and the amount of liquid from the gas subsystem to the liquid subsystem

(27) $$\begin{array}{l} h_{L}={\left[\;h_{\mathrm{lG}}\;0\;\;0\;\right]}^{T}\\ h_{G}={\left[0\;h_{\mathrm{gL}}\;0\;\right]}^{T} \end{array}$$

A solution for this matter is using Jacobian matrix F to U as follows:

(28) $$\frac{\partial U}{\partial t}=Q+h-A\frac{\partial U}{\partial s},\;A=\frac{\partial F}{\partial U}$$

Due to the existence of two dependent variables in F and U, it is a hard to use Jacobian, then in order to simplify the mathematical operation; one set of preliminary variables will be defined. Here, liquid volume fraction, pressure and velocity are selected as preliminary variables which are not unique. The point that must be taken into account is that in this formula, we have ${\text{A}}_{\text{U}}^{-1}$ and if just for one repetition, we have zero determinant, the existing matrix is not reversible and no estimation will be done. To avoid having zero determinant and to overcome this problem, a new idea is presented and the main states are extracted based on primitive variables:

(29) $$O=\left[\begin{array}{c} O_{1}\\ O_{2}\\ O_{3} \end{array}\right]=\left[\begin{array}{c} \rho_{l}\alpha_{l}\\ \rho_{g}\;\left(1-\alpha_{l}\right)\\ \rho_{m}\;u \end{array}\right]$$

(30) $$v=\left[\begin{array}{c} \frac{O_{2}}{1-\;\frac{O_{1}}{\rho_{l}}}\\ \frac{O_{3}}{O_{1}+O_{2}}\\ \frac{O_{1}}{\rho_{l}} \end{array}\right]=\left[\begin{array}{c} \rho_{g}\\ u_{g}\\ \alpha_{g} \end{array}\right]$$

(31) $$v={\left(\rho_{g}\;\;u_{g}\;\;\alpha_{l}\right)}^{T}$$

Changing the variables and defining new matrixes, equation form will be changed to the new following form:

(32) $$\frac{\partial U}{\partial v}\frac{\partial v}{\partial t}+\frac{\partial F}{\partial v}\frac{\partial v}{\partial s}=\text{Q}+\text{h}$$

The detail calculations of the new form of equations are presented in Appendix. A:

The decentralized system equations will be as follows:

(33) $$for\;liquid\;subsystem\{\begin{array}{c} \frac{\partial v}{\partial t}=-{\tilde{A}}_{L}\frac{\partial v}{\partial s}+{\tilde{Q}}_{L}+{\tilde{h}}_{L}\\ y_{L}=Cv \end{array}$$

(34) $$for\;gas\;subsystem\{\begin{array}{c} \frac{\partial v}{\partial t}=-{\tilde{A}}_{G}\frac{\partial v}{\partial s}+{\tilde{Q}}_{G}+{\tilde{h}}_{G}\\ y_{G}=Cv \end{array}$$

Following the fact that in real existing process in the refinery, the outlet measured signals and system variables are exposed to disturbance. W1 and W2 are constant weight matrixes and ω is the disturbance vector and it is limited as $\omega \left(t\right)\in l_{2}^{s}$ and by adding disturbance to Eqs. (33) and (34), the general from of the equations become as follows:

(35) $$S_{i}:\{\begin{array}{c} \frac{\partial v_{i}}{\partial t}=-{\tilde{A}}_{i}\frac{\partial v_{i}}{\partial s}+{\tilde{Q}}_{i}+{\tilde{h}}_{i}\left(v\left(t\right)\right)+W_{1}\omega \left(t\right)\\ \;\;y_{i}=C_{i}v_{i}\left(t\right)+W_{1}\omega \left(t\right) \end{array},\;i=1,2$$

## 4 Observer design

Here, based on system model, nonlinear observer is designed, first without considering disturbance and then by considering disturbance that affects dynamic and output signals. Most of the researches performed in the field of decentralized systems are by realizing the following inequalities and considering the interconnections between subsystems h_i_(v(t)) as an indefinite function. One of the important things that must be considered is the Jacobean limitation of this function that can be defined as follows [26]:

(36) $$a_{i}\leq \frac{\partial h_{i}\left(v\left(t\right)\right)}{\partial v}\leq b_{i}.\;c_{i}\leq \frac{\partial Q_{i}\left(v\left(t\right)\right)}{\partial v}\leq d_{i}$$

$${\text{a}}_{i}=min_{z\in R^{n}}(\frac{\partial h_{i}\left(v\left(t\right)\right)}{\partial v}\left(z\right),\;\;b_{i}=max_{z\in R^{n}}(\frac{\partial h_{i}\left(v\left(t\right)\right)}{\partial v}\left(z\right))$$

$$c_{i}=min_{z\in R^{n}}(\frac{\partial Q_{i}\left(v\left(t\right)\right)}{\partial v}\left(z\right),\;\;d_{i}=max_{z\in R^{n}}(\frac{\partial Q_{i}\left(v\left(t\right)\right)}{\partial v}\left(z\right))$$

This inequality emphasizes that h(v(t)) is αi Lipchitz and Q(v(t)) is β_i_ Lipchitz, such that: $\sqrt{\overset{2}{\underset{i=1}{\sum }}\text{max}({\left|c_{i}\right|}^{2}.{\left|d_{i}\right|}^{2}})\;\alpha_{i}=\sqrt{\overset{2}{\underset{i=1}{\sum }}\text{max}({\left|a_{i}\right|}^{2}.{\left|b_{i}\right|}^{2}})$

The total system will be as follows:

(37) $$S_{i}:\{\begin{array}{c} \frac{\partial v}{\partial t}=-\bar{A}{\dot{v}}_{s}+\tilde{Q}\left(v\left(t.s\right)\right)+\tilde{H}\left(v\left(t.s\right)\right)+W_{1}\omega \left(t\right)\\ y=Cv(t.s))+W_{2}\omega (t) \end{array}$$

where $y^{T}=\left(y_{1}^{T}.y_{2}^{T}\right)$, $\bar{A}=diag\left\{{\tilde{A}}_{1}.{\tilde{A}}_{2}\right\}$, $C=diag\left\{C_{1}.C_{2}\right\}$, ${H\left(v\left(t\right)\right)=\left\{{\tilde{h}}_{1}^{T}\left(v(t.s)\right).{\tilde{h}}_{2}^{T}\left(v\left(t.s\right)\right)\right\}}^{T}$, ${Q\left(v\left(t\right)\right)=\left\{{\tilde{Q}}_{1}^{T}\left(v(t.s)\right).{\tilde{Q}}_{2}^{T}\left(v\left(t.s\right)\right)\right\}}^{T}$ .$H\left(v_{i}\right):R^{n}\to R^{n}$, $Q\left(v_{i}\right):R^{n}\to R^{n}$ and it is assumed that they are differentiable versus v.

Using the matrixes and the general equations, the observer form is as follows:

(38) $$\{\begin{array}{c} \frac{\partial {\hat{v}}_{i}}{\partial t}=-{\tilde{A}}_{i}\frac{\partial {\hat{v}}_{i}}{\partial s}+{\tilde{H}}_{i}\left(v\left(t\right)\right)+{\tilde{Q}}_{i}\left(v\left(t\right)\right)+L_{i}\left(y_{G}-{\hat{y}}_{G}\right)\\ \;\;{\hat{y}}_{i}=C_{i}{\hat{v}}_{i}\left(t\right)\;\;i=1.\;2 \end{array}$$

The initial conditions are as follows:

$${\hat{v}}_{i}\left(s.t=0\right)={\hat{V}}_{i}^{0},\;{\hat{v}}_{i}\left(s=0.t\right)=0$$

where ${\hat{v}}_{i}$ is the state estimation and L is the observer gain matrix. As mentioned earlier in this system, no control action is done and controller optimization is not followed and observer optimization is followed in such a way that estimation error converges to zero which means $\text{ε}=v-\hat{v}\to 0$.

According to the existing interconnection between subsystems, the estimated error of dynamic is as follows:

(39) $$\dot{\text{ε}}=\left(\text{A}-\text{LC}\right)\cdot \text{ε}+\text{H}\left(\text{v}\left(\text{t}\right)\right)+\left({\text{W}}_{1}-{\text{LW}}_{2}\right)\text{ω}\left(\text{t}\right)$$

where, $Q=Q\left(v\right)-Q\left(\hat{v}\right)$

Considering the aforementioned formula, the augmented system will be as follows:

(40) $$\begin{array}{l} \left[\begin{array}{c} \dot{v}\\ \dot{\varepsilon } \end{array}\right]=\left[\begin{array}{cc} A & 0\\ 0 & A-LC \end{array}\right]\left[\begin{array}{c} v\\ \varepsilon \end{array}\right]+\left[\begin{array}{c} I_{n}\\ I_{n} \end{array}\right]Q\left(v\left(t\right)\right)+\left[\begin{array}{c} I_{n}\\ I_{n} \end{array}\right]h\left(v\left(t\right)\right)+\left[\begin{array}{c} 0\\ I_{n} \end{array}\right]\Delta Q\left(v\left(t\right)\right)+\left[\begin{array}{c} {\text{W}}_{1}\\ {\text{W}}_{1}-{\text{LW}}_{2} \end{array}\right]\omega \left(t\right)\;\\ v=\text{Λ}\tilde{v} \end{array}$$

where it can be shown that:

(41) $$\begin{array}{c} \begin{array}{c} \begin{array}{c} \overset{˙}{\tilde{v}}=\tilde{A}\tilde{v}+\Sigma Q\left(\text{v}\left(\text{t}\right)\right)+\Pi \Delta Q\left(\text{v}\left(\text{t}\right)\right)+\Gamma h\left(\text{v}\left(\text{t}\right)\right)+W\omega (t)\\ \Gamma =\left[\begin{array}{c} I_{n}\\ I_{n} \end{array}\right]\\ \Sigma =\left[\begin{array}{c} I_{n}\\ 0 \end{array}\right]\\ \Pi =\left[\begin{array}{c} 0\\ I_{n} \end{array}\right] \end{array}\\ \text{Λ}=\left[\begin{array}{cc} I_{n} & 0 \end{array}\right] \end{array}\\ W=\left[\begin{array}{c} {\text{W}}_{1}\\ {\text{W}}_{1}-{\text{LW}}_{2} \end{array}\right] \end{array}$$

To ensure asymptotical stability of system, the new LMI is calculated to achieve observer gain L.

## 5 Performance analysis

For stability analysis, we use Lyapunov method and define the following normal Lyapunov function:

(42) $$V\left(\tilde{v}\right)={\tilde{v}}^{T}P\tilde{v}$$

Where Lyapunov matrix P is as follows:

(43) $$P=\left[\begin{array}{cc} P_{s} & 0\\ 0 & P_{0} \end{array}\right]$$

where $P_{0}=P_{0}^{T}=\mathrm{diag}\left\{P_{0i}\right\}$ and $P_{s}=P_{s}^{T}=\mathrm{diag}\left\{P_{\mathrm{si}}\right\}$ are Lyapunov matrixes which are definite positive and it confirms the positivity of $\text{V}\left(\tilde{\text{v}}\right)$.

The issue is divided in to two parts: first we will analyze stability condition without the existence of disturbance and then we will consider the presence of disturbance. First, we consider that disturbance is zero where ω(t)=0 and for realizing this condition, we will survey $\frac{d}{\mathrm{dt}}V\left(\tilde{v}\right)<0$.

(44) $$\frac{d}{dt}V\left(\tilde{v}\right)={\left(\tilde{A}\tilde{v}+\tilde{Q}+\text{Δ}\tilde{Q}+\tilde{H}\right)}^{T}P\tilde{v}+{\tilde{v}}^{T}P\left(\tilde{A}\tilde{v}+\tilde{Q}+\text{Δ}\tilde{Q}+\tilde{H}\right)<0$$

This results in:

(45) $${\tilde{v}}^{T}\left({\tilde{A}}^{T}P+P\tilde{A}\right)\tilde{v}+{\tilde{H}}^{T}P\tilde{v}+{\tilde{v}}^{T}P\tilde{H}+{\tilde{Q}}^{T}P\tilde{v}+{\tilde{v}}^{T}P\tilde{Q}+\text{Δ}{\tilde{Q}}^{T}P\tilde{v}+{\tilde{v}}^{T}P\text{Δ}\tilde{Q}<0$$

So if ${\text{Ε}}_{z}=\left[{{\text{Ε}}_{z}^{T}}_{1}.\;\ldots .\;{{\text{Ε}}_{z}^{T}}_{N}\;\right]$, based on definition 1 in Appendix B, the vectors H, Q and ΔQ can be written as follows:

(46) $$h\left(v\left(t\right)\right)=E_{z}^{H}v,\;Q\left(v\left(t\right)\right)=E_{z}^{Q}v,\;\Delta Q\left(v\left(t\right)\right)=E_{z}^{\text{Δ}Q}$$

Remark: As shown in Formula 45, these sentences ${\tilde{H}}^{T}P\tilde{v}+{\tilde{v}}^{T}P\tilde{H}$ do not have square form and for this reason a transform function is used to change to a square inequality form, so for this reason, by using Eq. (41), we will have:

(47) $$\tilde{H}={\text{Ψ}}^{H}\tilde{v},\;\tilde{Q}={\text{Ψ}}^{Q}\tilde{v}$$

where:

(48) $${\text{Ψ}}^{H}={\text{ΓΕ}}_{z}^{HT}\text{Λ}.\;{\text{Ψ}}^{Q}={\text{ΓΕ}}_{z}^{QT}\text{Λ}$$

Based on Eq. (45) and the existing propositions in [36], the asymptotical stability conditions will become as follows: ${\tilde{v}}^{T}\left({\tilde{A}}^{T}P+P\tilde{A}+{\text{Ψ}}^{QT}P+P{\text{Ψ}}^{Q}+{\text{Ψ}}^{HT}P+P{\text{Ψ}}^{H}\right)\tilde{v}<0$

(49) $$\tilde{A}=\left[\begin{array}{cc} A & 0\\ 0 & A+E_{\text{Δ}Q}-LC \end{array}\right]$$

**Theorem 1** [37]:estimation error is asymptotically stable if there exist matrices $P\;=P^{T}>0$ and R of appropriate dimensions such that the following LMI is feasible:

$$Block\;Diagonal\left(H\left(\alpha^{1}\right).H\left(\alpha^{2}\right).\cdots .H\left(\alpha^{2qn}\right)\right)<0.\alpha^{j}\in X_{Hq.n}$$

where:

(50) $$\left(H\left(\alpha^{j}\right)=A^{T}\left(\alpha^{j}\right)P-C^{T}R+PA\left(\alpha^{j}\right)-R^{T}C\right)$$

With this condition, LMI is feasible and observer optimization equals $\text{L}={\text{P}}^{-1}{\text{R}}^{\text{T}}$.

**Theorem 2:** In using Theorem 1, global system is stable in the sense of Lyapunov if there exist $P=P^{T}$ and observer gain $L=diag\left(L_{1},\ldots ,L_{N}\right)$ with appropriate dimensions exists such that the following LMI is feasible:

(51) $$Diag\left(F\left(\gamma^{1}.\beta^{1}\right).\ldots F\left(\gamma^{2^{N_{nn}}}.\beta^{2^{N_{nn}}}\right)\right)<0.\gamma^{i}\in V_{M_{n.n}^{N}}.\beta^{i}\in V_{W_{n.n}^{N}}$$

(52) $$\left(\gamma^{i}.\beta^{i}\right)={\tilde{A}}^{T}P+P\tilde{A}+{{\text{Ψ}}^{Q}}^{T}\left(\gamma^{i}\right)P+P{\text{Ψ}}^{Q}\left(\gamma^{i}\right)+{{\text{Ψ}}^{H}}^{T}\left(\beta^{i}\right)P+P{\text{Ψ}}^{H}\left(\beta^{i}\right)$$

To ensure if F<0, the following LMI must be accessible:

(53) $$\left[\begin{array}{cc} X_{11} & X_{12}\\ X_{21} & X_{22} \end{array}\right]<0$$

where:

(54) $$X_{11}=A^{T}P_{s}+P_{s}A+{E_{z}^{H}}^{T}P_{s}+P_{s}E_{z}^{H}+{E_{z}^{Q}}^{T}P_{s}+P_{s}E_{z}^{Q}$$

(55) $$X_{12}={E_{z}^{H}}^{T}P_{0}$$

(56) $$X_{21}=P_{0}E_{z}^{H}$$

(57) $$X_{22}=A^{T}P_{0}+P_{0}A-C^{T}L^{T}P_{0}-P_{0}\mathrm{LC}+{E_{z}^{\Delta Q}}^{T}P_{0}+P_{0}E_{z}^{\Delta Q}$$

By multiplying the two sides of this inequality (Eq. (53)) by the following matrix and changing the variable $\text{Z}={\text{P}}_{0}\text{L}$, we will have:

(58) $$\left[\begin{array}{cc} S & 0\\ 0 & I \end{array}\right],\;\;S=S^{T}=P_{s}^{-1}>0$$

As a result, the inequality (Eq. (53)) will be as follows:

(59) $$\left[\begin{array}{cc} SA^{T}+\mathrm{AS}+S{Ε}_{z}^{T}+{Ε}_{z}S & S{Ε}_{z}^{T}P_{0}\\ P_{0}{Ε}_{z}S & A^{T}P_{0}+P_{0}A-C^{T}Z^{T}-\mathrm{ZC} \end{array}\right]$$

Based on the proven Theorem 4.1 in [37], if LMI becomes feasible, by solving this, the observer optimization becomes $\text{L}={\text{P}}_{0}^{-1}\text{Z}$.

Scenario 2: We assumed that ω(t)≠0 which means that the system is considered in the presence of disturbance. For the proposed system model in (37) and designed observer in (38), this problem changes to ${\text{H}}_{\infty }$ observer design which leads to the determination of observer gain L in a way that the estimation error converges to zero [14]. In other words:

(60) $$\underset{t\to \infty }{\lim}\varepsilon \left(t\right)=0\;\;\mathrm{for}\;\omega \left(t\right)=0$$

(61) $$\varepsilon_{L_{2}^{s}}\leq \lambda \omega_{L_{2}^{s}}\;\;\mathrm{for}\;\omega \left(t\right)\neq 0\;\mathrm{and}\;\;\varepsilon \left(0\right)=0$$

where λ>0 is the scalar number which shows the disturbance attenuation level. To provide Eqs. (60) and (61), we must find Lyapunov function as follows:

(62) $$\dot{v}+\varepsilon^{T}\varepsilon -\lambda^{2}\omega^{T}\omega <0$$

To ensure Eq. (62) is acquired from Eqs. (60) and (61) is too simple. By integrating both sides of Eq. (48), we will have:

(63) $$V\left(t_{f}\right)<V\left(0\right)-\overset{t_{f}}{\underset{0}{\int }}\varepsilon^{T}\left(\theta \right)\varepsilon \left(\theta \right)d\theta +\lambda^{2}\overset{t_{f}}{\underset{0}{\int }}\omega^{T}\left(\theta \right)\omega \left(\theta \right)d\theta$$

By considering that for all time span, v(t)≥0, ${\left\|\text{ε}\right\|}_{{\text{L}}_{2}}\leq \text{λ}{\left\|\text{ω}\right\|}_{{\text{L}}_{2}}$will be confirmed. The presented system in Eq. (37) and designed observer in Eq. (38) are accessible, if P and Z are found appropriately, then the following LMI is possible:

Thus, the mentioned LMI will be achieved in the following way:

(64) $$\left[\begin{array}{cc} {\tilde{A}}^{T}P+P\tilde{A}+{\text{Ψ}}^{T}P+P\text{Ψ}+\text{Ι} & P\left(W_{1}+LW_{2}\right)\\ \left(W_{1}^{T}+L^{T}W_{2}^{T}\right)P & -\lambda^{2}\text{Ι} \end{array}\right]$$

It can observed that in this condition, LMI is also feasible by using Theorem 1 and solving that the observer gain will be $\text{L}={\text{P}}_{0}^{-1}\text{Z}$ .

## 6 Unscented Kalman Filter

The Kalman filter is a recursive estimator, which estimates the states and parameters of a linear systems by integrating measured data in real-time. It is initially developed for linear systems but several extensions of that exist for nonlinear systems which Unscented Kalman filter (UKF) is one of the most popular extensions because of its ease of implementation and simple concept. One of the most common way of applying the Kalman Filter (KF) for a nonlinear system is the augmented Extended Kalman Filter (EKF) with using linearization and estimating both the state and parameter vectors of the system. The EKF needs the Jacobian matrices which is difficult to obtain for higher order systems. Further, the linearization may introduce errors in the state estimation which may lead the state to diverge over time [38]. The Unscented Kalman Filter principle is simple and easy to implement as it does not require the calculation of Jacobian at each time step [39]. In [40, 41], it is shown that for nonlinear systems the unscented Kalman filter (UKF) has a better performance than other Kalman filter types. In UKF the propagation of the mean and covariance matrix of the estimation error is done through nonlinear transformations and sample points which are termed as sigma points around the mean value by using a deterministic sampling approach known as the unscented transformation [42].

The augmented state vector is defined by $x^{a}=\;\left[X,\theta \right]$ where X is the state and θ is the parameters of the model.

Each sigma point is propagated through the nonlinear process so that the augmented state vector for the discrete time step k is written as follows:

(65) $$\left[\begin{array}{c} X_{k}\\ \theta_{k} \end{array}\right]=\left[\begin{array}{c} f\left(X_{k-1},\theta_{k-1}\right)\\ \theta_{k-1} \end{array}\right]+q_{k}=f^{a}\left(X_{k-1}\theta_{k-1}\right)+q_{k}$$

where $q_{k}∼N\left(0,Q_{k}\right)$ is the zero mean white Gaussian noise. Measurements and corrupting zero mean white noise for discrete-time model will be added as follows:

(66) $$\begin{array}{l} y_{k}=h\left(X_{k}\right)+r_{k}\\ \;r_{k}∼N\left(0,R_{k}\right) \end{array}$$

The process noise covariance matrix ($Q_{k}$) of UKF for system model and measurement noise covariance matrix ($R_{k}$) are as follows:

(67) $$\begin{array}{l} \text{Q}=\text{diag[}5\times 10^{-9}\;8\times 10^{-9}\text{]}\\ \text{R}=\text{diag[}0.8\times .0.5^{2}\;0.8\times .0.3^{2}\text{](}{\text{bar}}^{2}\text{)} \end{array}$$

Choosing the ($Q_{k}$) specifies trade-offs in the UKF design. Choosing larger ($Q_{k}$) leads to faster convergence but typically more error in the estimation and choosing smaller ($Q_{k}$) leads to slower convergence but typically less error in the estimation.

## 7 Simulation

In this article, measurements, data and information are acquired from a real process of a gas refinery in the POGC in Iran which are fed to HYSYS simulator. Because the results obtained from the DMVT simulation are time dependent and multiphase flow averaging over 24 h is constant, the averaged data is basically not suitable to determine the accuracy of the model thus getting this data from the refinery to prove the accuracy of the code is not the right justification. Therefore we need to provide another type of data that can validate the results of observer simulation, so the performance of the nonlinear observers based on DMVT model is evaluated against UKF based on a simplified model by using measurements from the HYSYS simulator.

**Figure 3:**
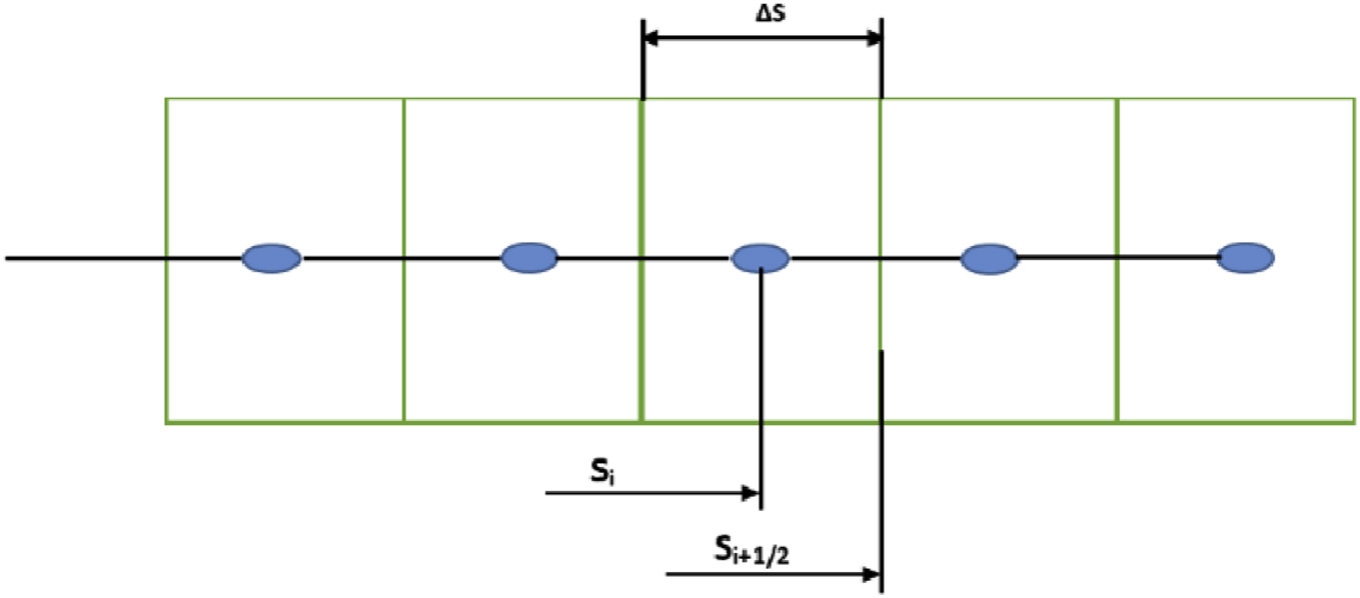
Discretization of spatial domain.

The upwind scheme has been used for numerical solution. The use of the upwind discretization leads to a numerical scheme which is first order accurate in space. But for more accuracy we have used second order upwind scheme. Schematic of discretization is added in the article. To perform numerical solution of the drift flux model in the form of Eqs. (3) and (4), first, we discretized the spatial domain into a finite number of control volumes without overlapping between them. The mentioned differential equations are integrated over each control volume as Figure 3. Cell centers and length of the grid blocks of mesh is as follows:

(68) $$Centers:S_{i}=0.5\left(S_{i+1/2}+S_{i-1/2}\right)$$

(69) $$Length:\Delta S_{i}=S_{i+1/2}-S_{i-1/2}$$

The values of the variables are located at the center of cells so $v_{i}=v\left(S_{i}\right)$ is the $i^{th}$ block variables, so for each subsystem we have 24 nodes with a time step of 0.01second via 100 m length of the pipes. Pipes material is carbon steel ASTM A106 Seamless. Simulation is performed by using MATLAB in parallel by HYSYS simulator. We have used HYSYS with real process data to check the performance of designed observer and run the MATLAB code using measurement from the dynamic HYSIS simulator. All parameters and variables used in the simulation are in line with the existing process in the refinery. In the Table 2, the related information about a real process is mentioned:

**Table 2:** Real process data for numerical solution.

Pipe roughness *(*m*10^−6^*)*	Gas Density *(*kg/m^3^*)*	Gas Viscosity *(Pa.s)*	Liquid Viscosity *(Pa.s)*	Liquid Density *(kg/m*^3^*)*	Gas pipe Diameter (mm)	Liquid pipe Diameter (mm)
45	12	1.9E-5	0.01	1000	457	203

**Figure 4:**
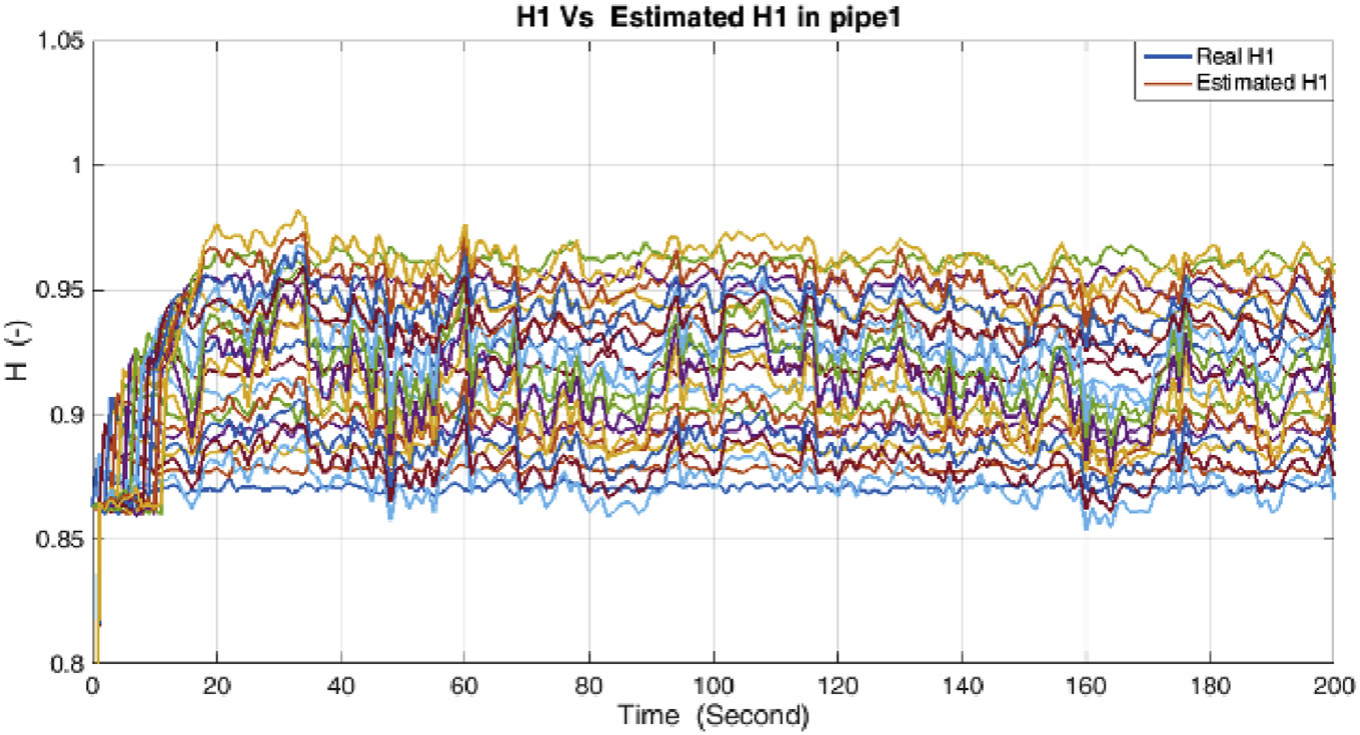
Liquid phase fraction and its estimation in the Liquid pipe.

**Figure 5:**
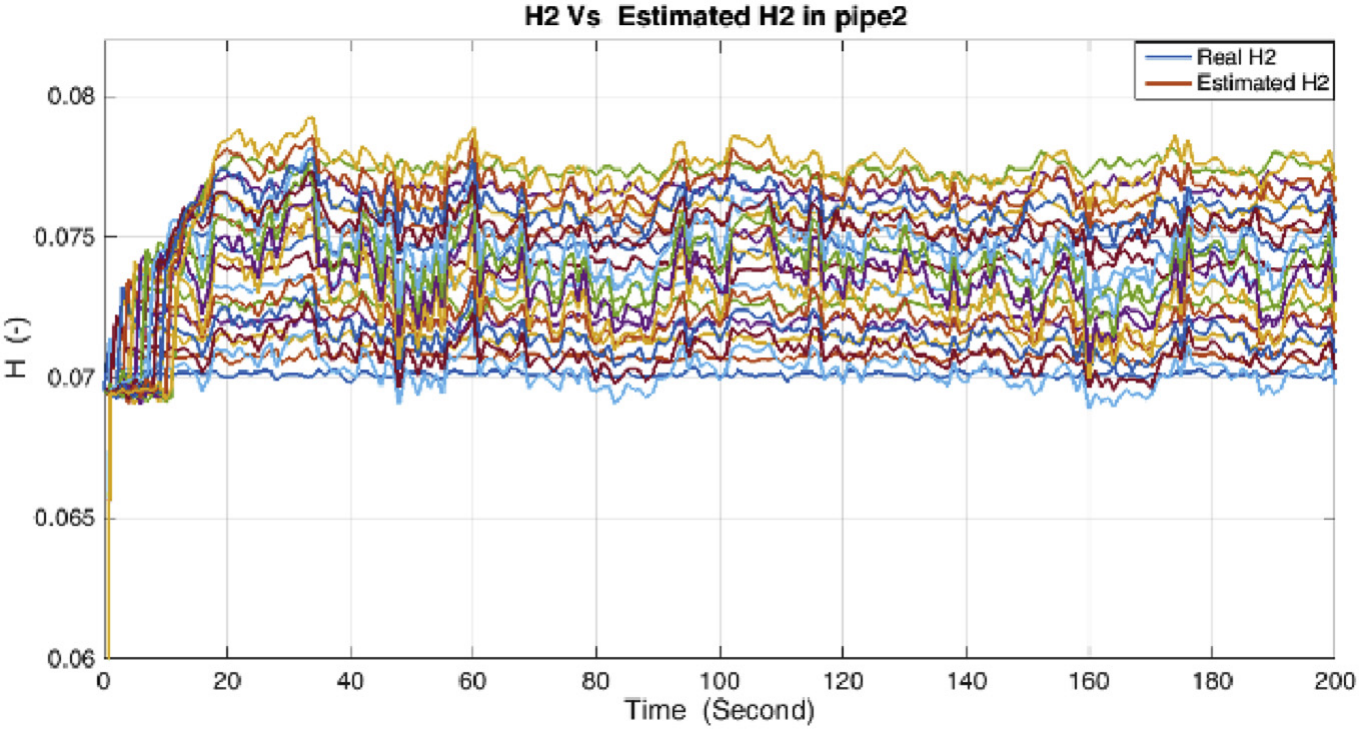
Liquid phase fraction and its estimation in the Gas pipe.

**Figure 6:**
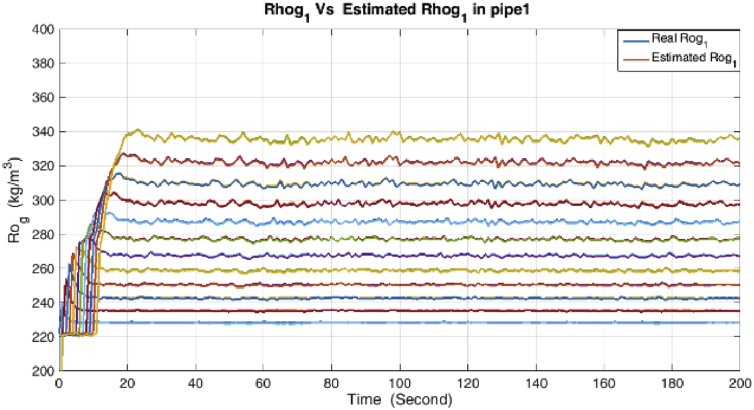
Density and its estimation in the condensate pipe.

**Figure 7:**
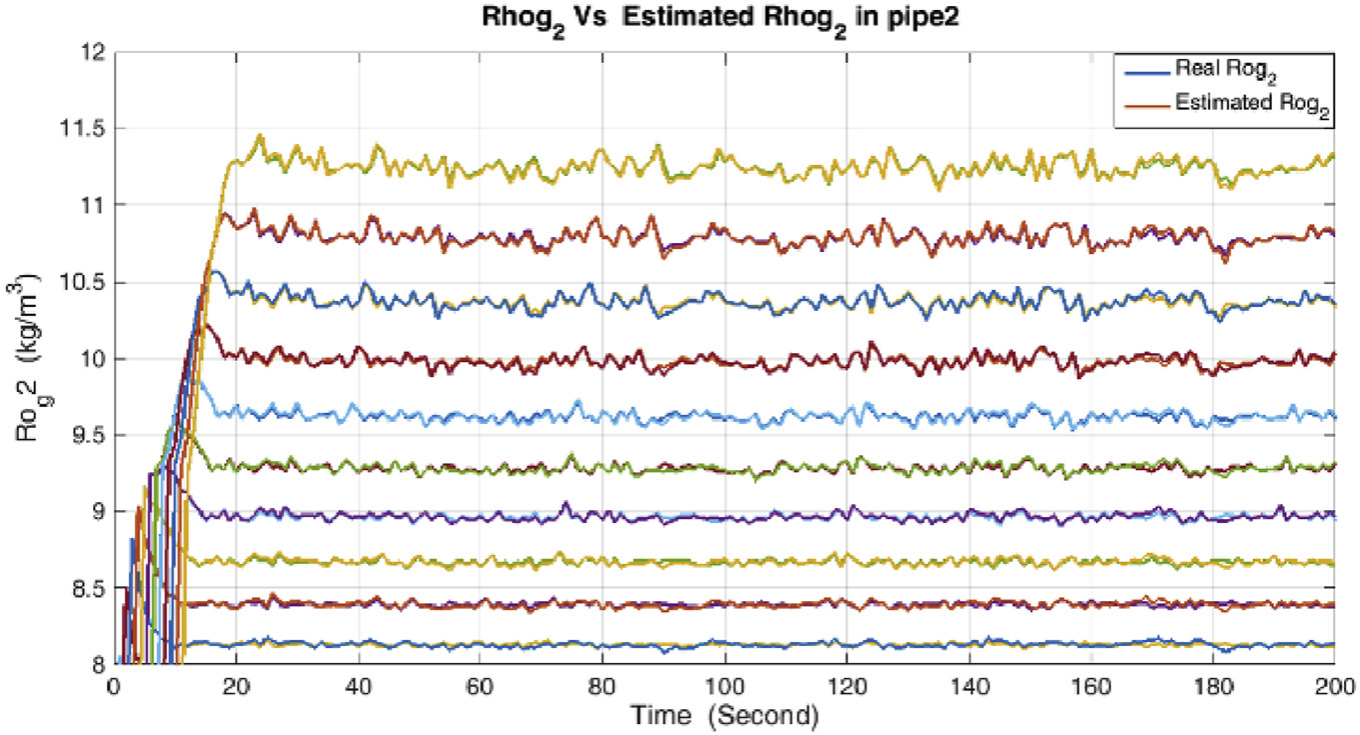
Density and its estimation in the Gas pipe.

**Figure 8:**
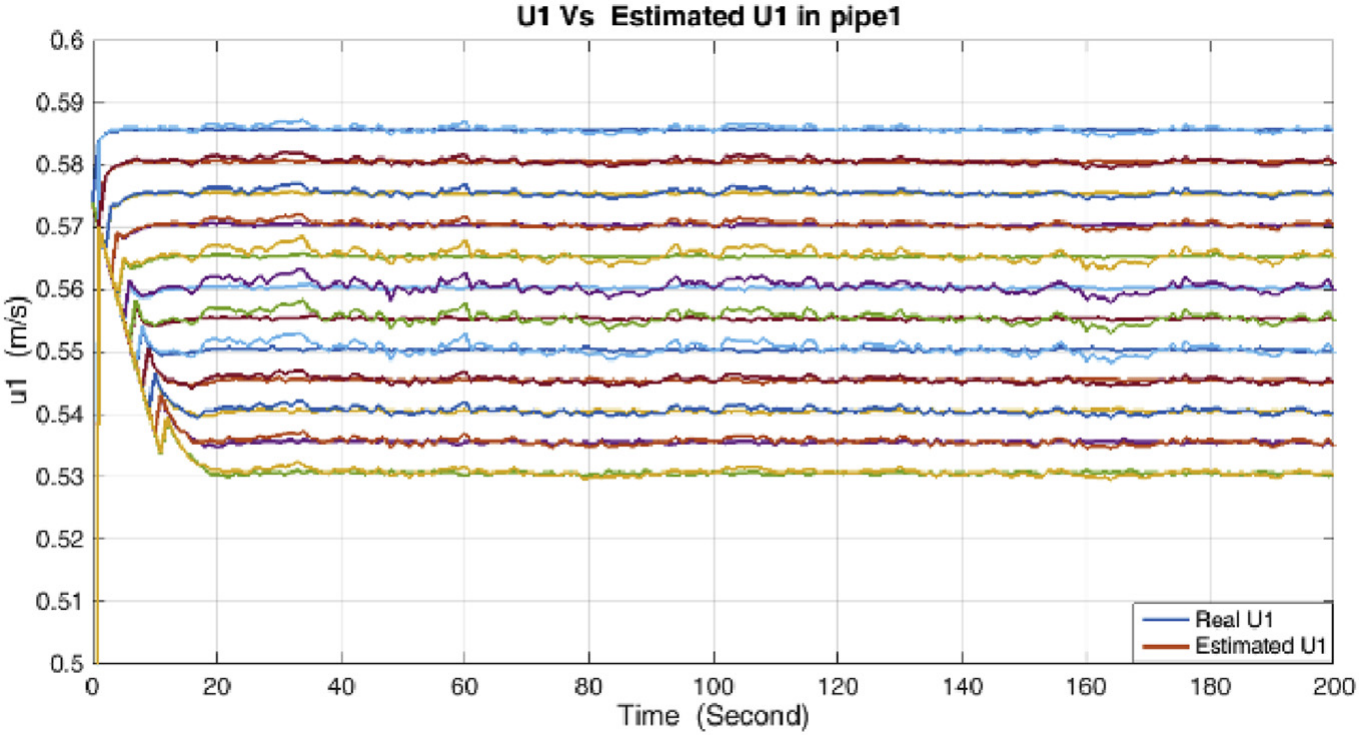
Velocity and its estimation in the condensate pipe.

**Figure 9:**
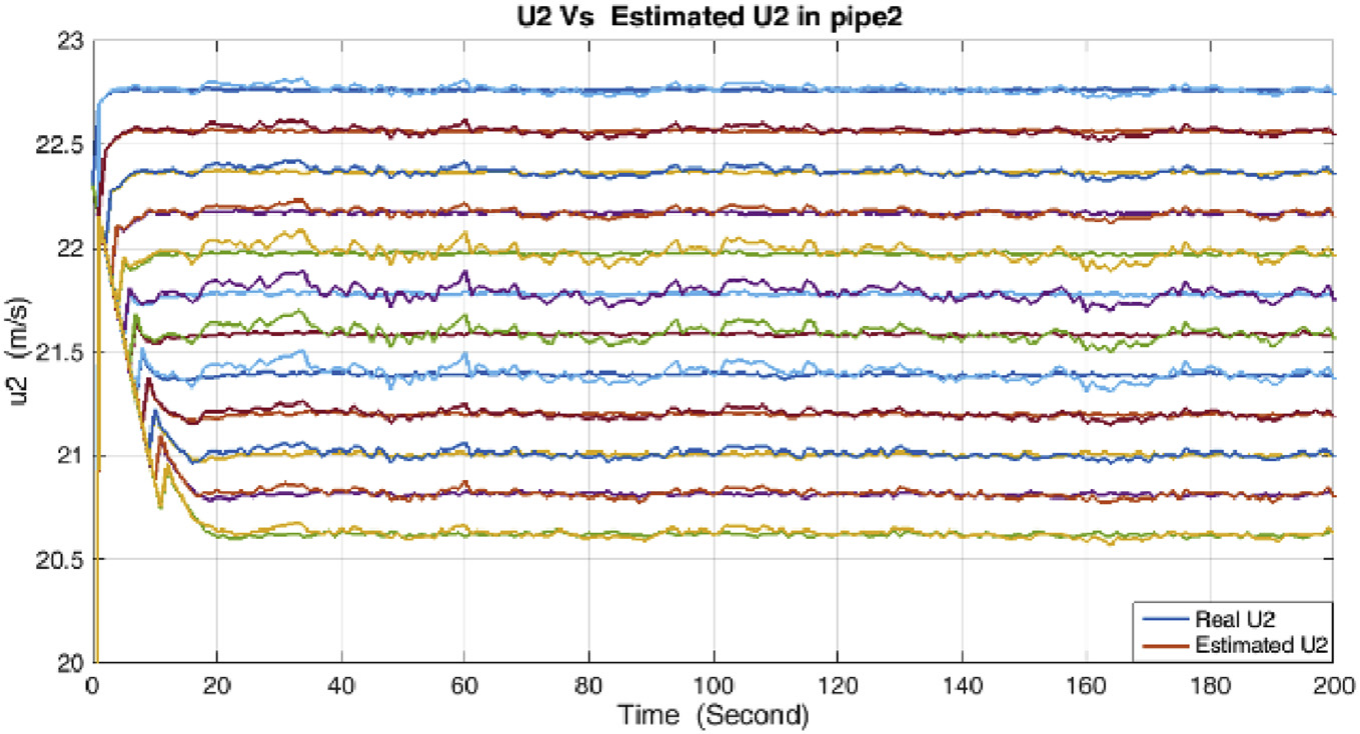
Velocity and its estimation in the Gas pipe.

**Figure 10:**
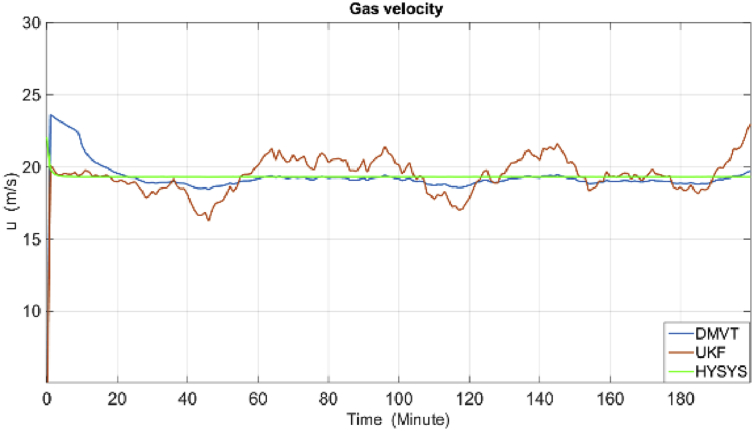
Gas line velocity at node 24.

**Figure 11:**
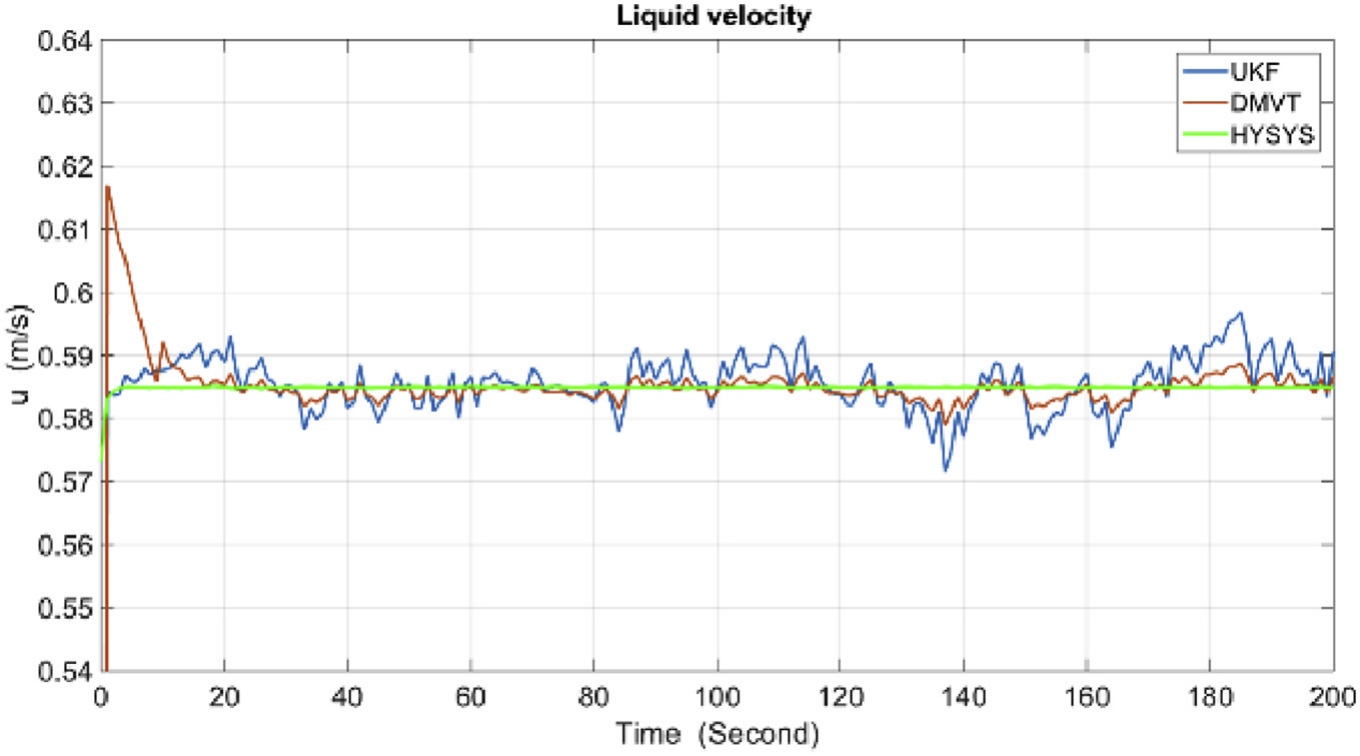
Condensate line velocity at node 24.

The results of simulations are as shown in the following figures. Figure 4 shows the real and estimated total state variables of both subsystems. Because of the high number of trends and figures, the liquid phase fraction, density and velocity are illustrated separately in Figures 4, 5, 6, 7, 8, and 9. So velocities in gas and condensate pipes at node 12 as essential variables in flow measurement are illustrated in Figures 10 and 11. Node 12 is chosen just as a sample without any importance.

As it is shown, all states are estimated with reasonable accuracy.

**Figure 12:**
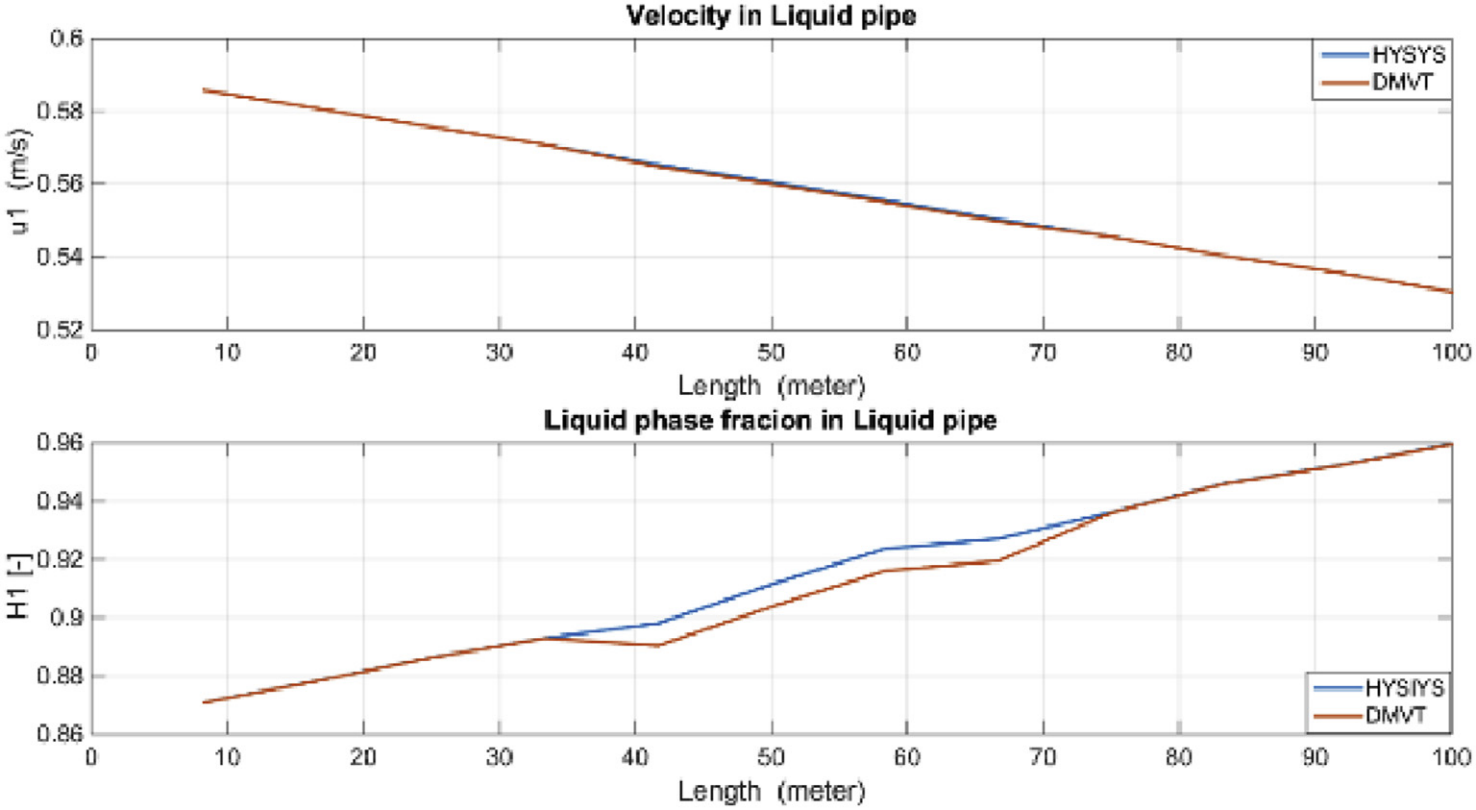
Liquid fraction and velocity in condensate line.

**Figure 13:**
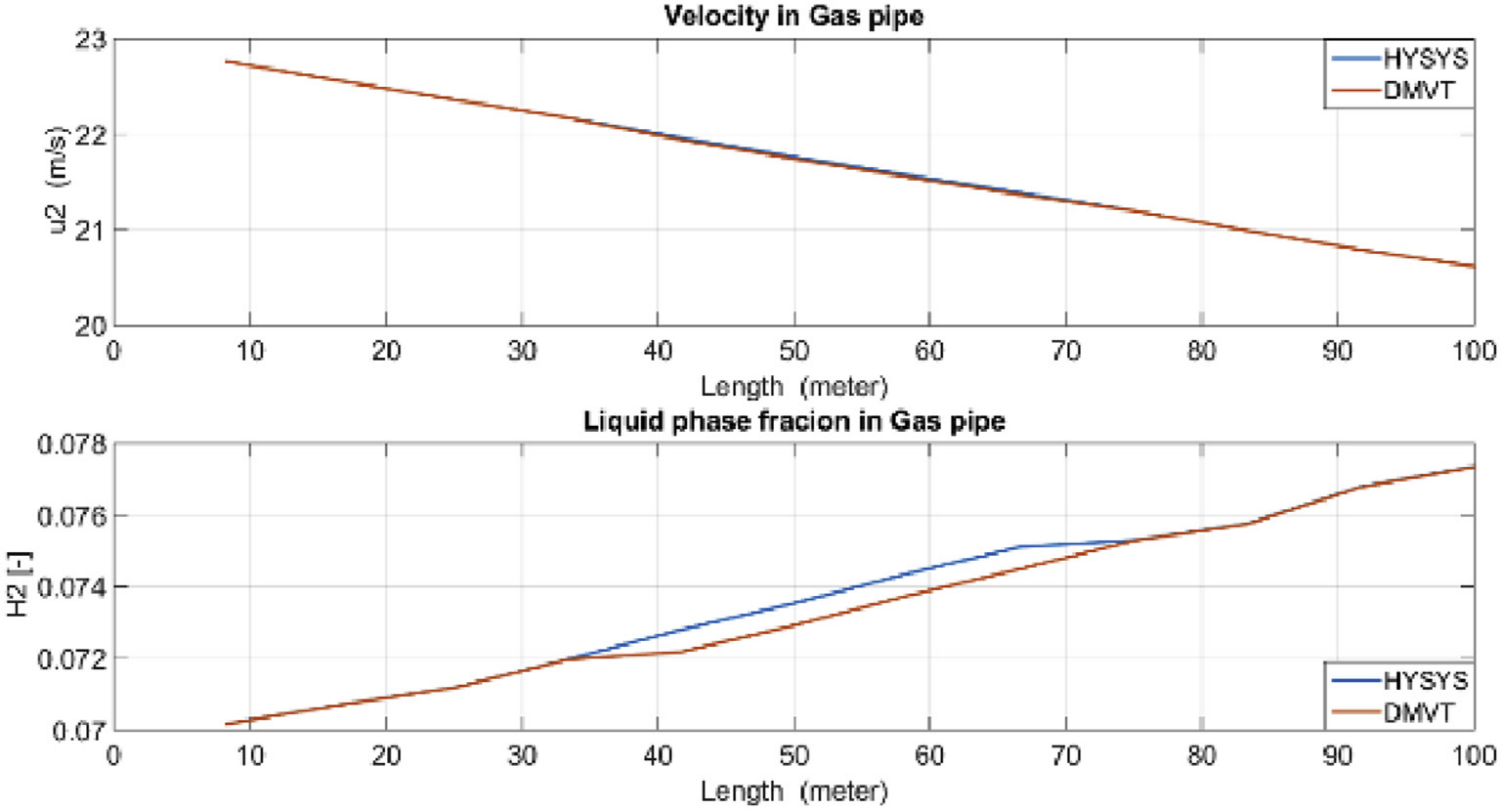
Liquid fraction and velocity in gas line.

Velocities and liquid fractions in gas and liquid pipes versus pipes length are illustrated in Figures 12 and 13. This results show that because of frictional losses, the velocity of fluid along the pipe length is reducing but liquid phase fraction is increasing. These variables are identified with reasonable accuracy by both estimators*.*

**Figure 14:**
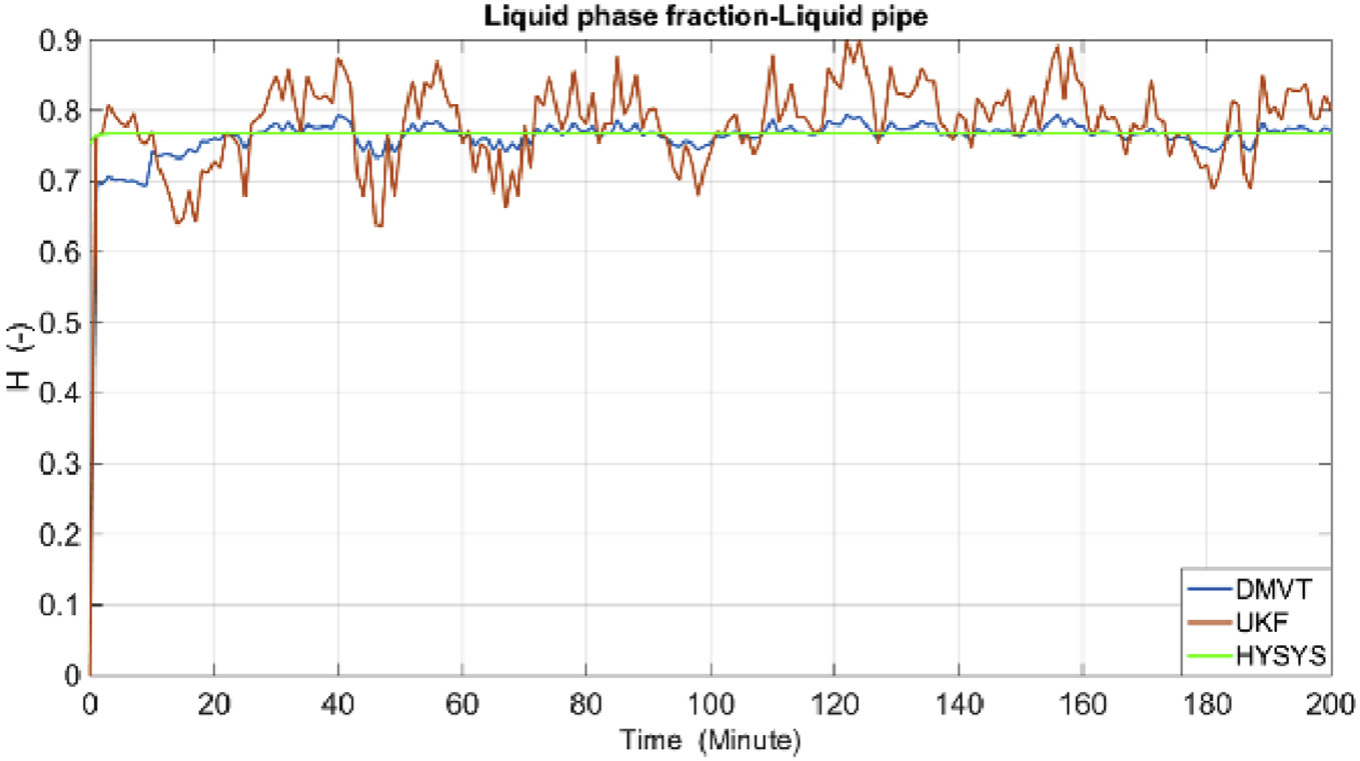
Liquid fraction in the condensate line.

**Figure 15:**
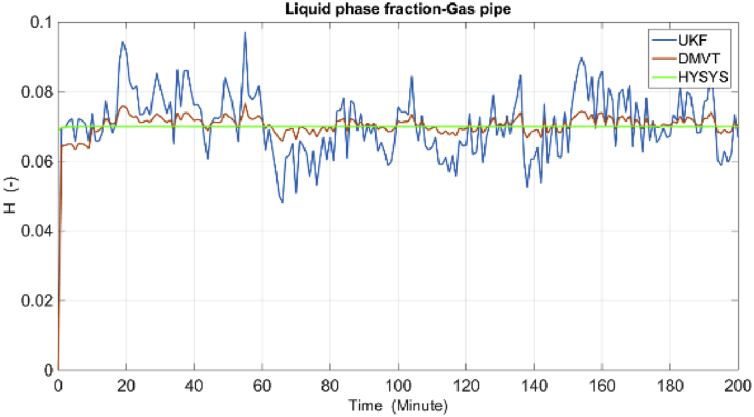
Liquid fraction in the gas line.

The estimation of the liquid phase fraction in the gas and condensate subsystems versus the pipes length are shown in Figures 14 and 15, respectively. The estimates of both algorithms are converging quite fast, less than 20 min but the time factor is not shown in these figures. The DMVT-based observer has better performance than the UKF for estimation of the liquid phase fraction*.*

HYSYS simulation was conducted based on this real process data, partially presented in Table 2. In this simulation, pressure control on the pipelines, as well as pressure and level control for storages were considered.

The RMSE metric for the DMVT-based observer and UKF for both models during the estimation period for the states in case of 1% error on the pipe pressure measurements, are summarized in Table 3.

**Table 3:** RMSE metric for states.

Method	Density	Velocity	Liquid Phase fraction
DMVT observer	$2.4\times 10^{-3}$	$2.8\times 10^{-3}$	$3.3\times 10^{-3}$
UKF	$3.8\times 10^{-3}$	$4.5\times 10^{-3}$	$5.4\times 10^{-3}$

According to the RMSE metric Table 3, the DMVT-based observer has better performance than UKF for estimation of the states. Since the point wise volumetric flow rate is obtained by multiplying the velocity to the cross-sectional area and by multiplying the result to the density, the mass flow rate is evaluated. So because the line pressure has a direct effect on the states which are directly related to the mass flow rates in the subsystems, small inaccuracies in the line pressure measurement have a significant effect on the estimation of flow rate. As is clear from the figures, UKF needs more time to reach its asymptotic level of accuracy, therefore, also its performance is satisfactory but it is lower than performance of proposed observer.

## 8 Conclusion

In this article, the simplified drift flux model (DFM) is used for multiphase fluids at the gas refinery entrance, as a decentralized system composed of two subsystems, and converted into conventional equations, based on which a decentralized nonlinear observer was designed considering the subsystem interconnections. Then, its stability was investigated using DMVT and converting this system into a LPV system and the conditions into linear matrix inequality (LMI). Finally, a disturbance with bonded norm was applied to the system dynamics and output.

The HYSYS simulation is used to obtain the required measurements and the result of this simulation will be a reference for comparing the performance of proposed methods.

Additionally, the Unscented Kalman filter (UKF) based on the simplified drift flux model (DFM) was used to estimate the states then both methods' results are compared with the HYSYS simulation using the real process data, it is found that both observers (DMVT based and UKF) are capable to detect and identify the states using a simulated scenario with HYSYS simulator with some differences in performance and drift flux model (DFM) model is sufficient for estimation of parameters and states of the multiphase flow entering the gas refinery.

Although the results show that all states are identified with reasonable accuracy by both estimators, the DMVT-based observer has better performance than the UKF based on DFM for estimation of the flow characteristics of multiphase flow but the proposed DMVT observer is more sensitive to errors in the refinery's output and parameters of the model than the UKF. The simulation results indicate high efficiency of both estimation methods without needing observer gain, suggesting that the proposed methods are suitable for stable operational conditions and have high reliability.

## Declarations

### Author contribution statement

Abolfazl Varvani Farahani & Mohsen Montazeri: Conceived and designed the experiments; Performed the experiments; Analyzed and interpreted the data; Contributed reagents, materials, analysis tools or data; Wrote the paper.

### Funding statement

This research did not receive any specific grant from funding agencies in the public, commercial, or not-for-profit sectors.

### Competing interest statement

The authors declare no conflict of interest.

### Additional information

No additional information is available for this paper.

## Appendix A PDE detail calculation

$$A_{U}=\frac{\partial U}{\partial v}\cdot A_{F}=\frac{\partial F}{\partial v}$$

$$\frac{\partial v}{\partial t}+A_{U}^{-1}A_{F}\frac{\partial v}{\partial s}=A_{U}^{-1}Q+A_{U}^{-1}h$$

$$\tilde{A}=A_{U}^{-1}A_{F}\cdot \tilde{Q}=A_{U}^{-1}Q,\;\tilde{h}=A_{U}^{-1}h$$

$$\frac{\partial v}{\partial t}+\frac{\tilde{A}\partial v}{\partial s}=\tilde{Q}+\tilde{h}$$

Based on Equations 13 to 19, Formula 27 will be as follows:

$$\{\begin{array}{c} A_{U_{L}}=\frac{\partial U_{L}}{\partial v}=\left[\begin{array}{ccc} 0 & 0 & \rho_{\mathrm{lL}}\\ \left(1-\alpha_{\mathrm{lL}}\right)/R_{\mathrm{gL}}T_{0} & 0 & -\rho_{\mathrm{gL}}\\ \rho_{\mathrm{gL}}u_{\mathrm{gL}}\left(1-\alpha_{\mathrm{lL}}\right)/R_{\mathrm{gL}}T_{0} & \rho_{\mathrm{gL}}\left(1-\alpha_{\mathrm{lL}}\right) & \rho_{\mathrm{lL}}u_{\mathrm{lL}}-\rho_{\mathrm{gL}}u_{\mathrm{gL}} \end{array}\right]\\ A_{U_{G}}=\frac{\partial U_{G}}{\partial v}=\left[\begin{array}{ccc} 0 & 0 & \rho_{\mathrm{lG}}\\ \left(1-\alpha_{\mathrm{lG}}\right)/R_{\mathrm{gG}}T_{0} & 0 & -\rho_{\mathrm{gG}}\\ \rho_{\mathrm{gG}}u_{\mathrm{gG}}\left(1-\alpha_{\mathrm{lG}}\right)/R_{\mathrm{gG}}T_{0} & \rho_{\mathrm{gG}}\left(1-\alpha_{\mathrm{lG}}\right) & \rho_{\mathrm{lG}}u_{\mathrm{lG}}-\rho_{\mathrm{gG}}u_{\mathrm{gG}} \end{array}\right] \end{array}$$

$$\{\begin{array}{c} A_{F_{L}}=\frac{\partial F_{L}}{\partial v}=\left[\begin{array}{ccc} 0 & 0 & \rho_{\mathrm{lL}}u_{\mathrm{lL}}\\ u_{\mathrm{gL}}\left(1-\alpha_{\mathrm{lL}}\right) & \rho_{\mathrm{gL}}\left(1-\alpha_{\mathrm{lL}}\right) & -\rho_{\mathrm{gL}}.u_{\mathrm{gL}}\\ 1+u_{\mathrm{gL}}^{2}\left(1-\alpha_{\mathrm{lL}}\right) & 2\rho_{\mathrm{gL}}.u_{\mathrm{gL}}.\left(1-\alpha_{\mathrm{lL}}\right) & \left(\rho_{\mathrm{lL}}u_{\mathrm{lL}}^{2}-\rho_{\mathrm{gL}}u_{\mathrm{gL}}^{2}\right) \end{array}\right]\\ A_{F_{G}}=\frac{\partial F_{G}}{\partial v}=\left[\begin{array}{ccc} 0 & 0 & \rho_{\mathrm{lG}}u_{\mathrm{lG}}\\ u_{\mathrm{gG}}\left(1-\alpha_{\mathrm{lL}}\right) & \rho_{\mathrm{gL}}\left(1-\alpha_{\mathrm{lL}}\right) & -\rho_{\mathrm{gG}}.u_{\mathrm{gG}}\\ 1+u_{\mathrm{gG}}^{2}\left(1-\alpha_{\mathrm{lG}}\right) & 2\rho_{\mathrm{gG}}.u_{\mathrm{gG}}.\left(1-\alpha_{\mathrm{lG}}\right) & \left(\rho_{\mathrm{lG}}u_{\mathrm{lG}}^{2}-\rho_{\mathrm{gG}}u_{\mathrm{gG}}^{2}\right) \end{array}\right] \end{array}$$

## Appendix B DMVT Application

Definition 1: [26] We assume $\text{Φ}{:\text{R}}^{\text{n}}\to R^{\text{n}}$ QUOTE and $\text{a.b}\in {\text{R}}^{\text{n}}$ QUOTE and also Φ that is differentiable on convex $C_{0}\left(a,b\right)$. If we have the S set as follows: $e_{s}\left(j\right)={\left(0,\ldots ,0,1,0,\ldots ,0\right)}^{T}\in R^{s},\;s\geq 1$

Then $e_{s}\left(j\right)={\left(0,\ldots ,0,1,0,\ldots ,0\right)}^{T}\in R^{s},\;s\geq 1$ exists for i=1,…,n in such a way that:

$\text{Φ}\left(a\right)-\text{Φ}\left(\text{b}\right)=\left[\overset{n,n}{\underset{i,j=1}{\sum }}e_{n}\left(i\right)e_{n}^{T}\left(j\right)\frac{\partial {\text{Φ}}_{i}}{\partial v_{j}}\left({\text{z}}_{i}\right)\right]\left(\text{a}-\text{b}\right)$ Based on Definition 1 and Theorem 9 in [26], it can be concluded that one Z vector exists as follows: $z_{j}^{H}\in C_{0}\left(v_{i},0\right),\;z_{j}^{Q}\in \;C_{0}\left(v_{i},0\right)\;\mathrm{and}\;\;z_{j}^{\Delta Q}\in C_{0}\left(v_{i},{\hat{v}}_{i}\right)\;\mathrm{for}\;\mathrm{all}\;j=1\ldots n$ Such that:

$$H_{i}\left({\text{v}}_{i}\right)-H_{i}\left(0\right)=\left(\overset{L,G}{\underset{j,k=G,L}{\sum }}e_{n}\left(j\right)e_{n}^{T}\left(k\right)\frac{\partial H_{i_{j}}}{\partial v_{k}}\left({\text{z}}_{j}^{H}\right)\right)\left({\text{v}}_{i}-0\right),Q_{i}\left(\text{v}\right)-Q_{i}\left(0\right)=\left(\overset{L,G}{\underset{j,k=G,L}{\sum }}e_{n}\left(j\right)e_{n}^{T}\left(k\right)\frac{\partial Q_{i_{j}}}{\partial v_{k}}\left({\text{z}}_{j}^{Q}\right)\right)\left({\text{v}}_{i}-0\right),Q_{i}\left({\text{v}}_{i}\right)-Q_{i}\left({\hat{\text{v}}}_{i}\right)=\left(\overset{L,G}{\underset{j,k=G,L}{\sum }}e_{n}\left(j\right)e_{n}^{T}\left(k\right)\frac{\partial Q_{i_{j}}}{\partial v_{k}}\left({\text{z}}_{j}^{\Delta Q}\right)\right)\left({\text{v}}_{i}-{\hat{\text{v}}}_{i}\right)$$

$$H_{i}\left(v_{i}\right)=E_{\mathrm{zi}}^{H}v_{i},\;\;Q_{i}\left(v_{i}\right)=E_{\mathrm{zi}}^{Q}v_{i},\;\;\Delta Q_{i}=E_{\mathrm{zi}}^{\Delta Q}\varepsilon_{i}$$

where:

$$\begin{array}{l} E_{\mathrm{zi}}^{H}=\overset{L,G}{\underset{j,k=G,L}{\sum }}e_{n}\left(j\right)e_{n}^{T}\left(k\right)\frac{\partial H_{\mathrm{ij}}}{\partial v}\left({{\text{z}}^{H}}_{j}\right)\\ E_{\mathrm{zi}}^{Q}=\overset{L,G}{\underset{j,k=G,L}{\sum }}e_{n}\left(j\right)e_{n}^{T}\left(k\right)\frac{\partial H_{\mathrm{ij}}}{\partial v}\left({{\text{z}}^{Q}}_{j}\right)\\ E_{\mathrm{zi}}^{\text{Δ}Q}=\overset{L,G}{\underset{j,k=G,L}{\sum }}e_{n}\left(j\right)e_{n}^{T}\left(k\right)\frac{\partial H_{\mathrm{ij}}}{\partial v}\left({{\text{z}}^{\Delta Q}}_{j}\right) \end{array}$$

So if ${\text{Ε}}_{z}=\left[{{\text{Ε}}_{z}^{T}}_{1},\ldots ,{{\text{Ε}}_{z}^{T}}_{N}\right]$ the vectors H, Q and ΔQ can be written as follows:

$$h\left(v\right)=E_{z}^{H}v,\;\;Q\left(v\right)=E_{z}^{Q}v,\;\;\Delta Q=E_{z}^{\Delta Q}\varepsilon$$
